# Mindfulness in sustainability science, practice, and teaching

**DOI:** 10.1007/s11625-017-0428-2

**Published:** 2017-04-05

**Authors:** Christine Wamsler, Johannes Brossmann, Heidi Hendersson, Rakel Kristjansdottir, Colin McDonald, Phil Scarampi

**Affiliations:** Lund University Centre for Sustainability Studies (LUCSUS), Lund, Sweden

**Keywords:** Ecological mindfulness, Organizational mindfulness, Political mindfulness, Compassion, Sustainability, Well-being, Contemplative teaching, Emotion, Planning, Risk reduction, Adaptation, Other ways of knowing, Spiritual ecology, Transformation, Inner transition

## Abstract

This paper explores the current role of mindfulness in sustainability science, practice, and teaching. Based on a qualitative literature review that is complemented by an experimental learning lab, we sketch the patterns and core conceptual trajectories of the mindfulness–sustainability relationship. In addition, we assess this relationship within the field of climate change adaptation and risk reduction. The results highlight that notions such as ‘sustainability from within’, ‘ecological mindfulness’, ‘organizational mindfulness’, and ‘contemplative practices’ have been neglected in sustainability science and teaching. Whilst little sustainability research addresses mindfulness, there is scientific support for its positive influence on: (1) subjective well-being; (2) the activation of (intrinsic/ non-materialistic) core values; (3) consumption and sustainable behavior; (4) the human–nature connection; (5) equity issues; (6) social activism; and (7) deliberate, flexible, and adaptive responses to climate change. Most research relates to post-disaster risk reduction, although it is limited to the analysis of mindfulness-related interventions on psychological resilience. Broader analyses and foci are missing. In contrast, mindfulness is gaining widespread recognition in practice (e.g., by the United Nations, governmental and non-governmental organizations). It is concluded that mindfulness can contribute to understanding and facilitating sustainability, not only at the individual level, but sustainability at all scales, and should, thus, become a core concept in sustainability science, practice, and teaching. More research that acknowledges positive emotional connections, spirituality, and mindfulness in particular is called for, acknowledging that (1) the micro and macro are mirrored and interrelated, and (2) non-material causation is part of sustainability. This paper provides the first comprehensive framework for contemplative scientific inquiry, practice, and education in sustainability.

## Introduction

Humanity is facing increasingly complex environmental and sustainability challenges (Kates et al. 2001; Sol and Wals 2015). They are a manifestation of what sustainability scientists describe as a “systemic world” characterized by multiple causations, interactions, complex feedback loops, and inevitable uncertainty and unpredictability (Lang et al. 2012). Issues such as climate change, disasters, energy, food, waste and water management, land use change, and biodiversity loss are highly complex and require an urgent response (Jerneck et al. 2011; Wals and Corocoran 2012).

Current coordination mechanisms, problem-solving strategies, and modes of scientific inquiry, teaching, and learning appear insufficient to address global sustainability challenges (Sol and Wals 2015). As a result, expanded consciousness, embodied in notions such as mindfulness, compassion and empathy, is emerging as a potential new area of exploration to address these challenges (Edwards 2015; Goleman 2011). Increasing research into mindfulness supports related advancements.

In fact, progress in neuroscience and neuroplasticity, described in both the scientific and popular literature, suggests that mindfulness can literally rewire our brains (Doty 2016; Hölzel et al. 2011; Lazar et al. 2005; Luders et al. 2009; Powietrzynska et al. 2015; Tang et al. 2012; Vestergaard-Poulsen et al. 2009), and may be a necessary component of the conversion to a more sustainable society (Koger 2015). Mindfulness is generally understood as intentional, compassionate, and non-judgmental attentiveness to the present moment (Baer 2003; Condon et al. 2013; Kabat-Zinn 1990), which is associated with greater emotional intelligence (Schutte and Malouff 2011).^1^ It is an inherent capacity of the human organism that is rooted in the fundamental activities of consciousness and linked to established theories of attention and awareness^2^ (Buss 1980). Its study is part of a longstanding field that recognizes the value of increased consciousness brought to bear on subjective experience, behavior, and the immediate environment (Amel et al. 2009; Brown et al. 2007; Carver and Scheier 1981; Csikszentmihalyi 1997; Duval and Wicklund 1972; Jacob et al. 2009).

As a strategy, mindfulness is increasingly used in various professional fields and disciplines ranging from health care and the performing arts to pedagogy and business (Black 2010; Boyce 2011). However, further research is needed to better understand the scope of such applications (Brown et al. 2007; Eriksen and Ditrich 2015). The question thus arises of whether the concept of mindfulness also applies to the sustainability field, at a time of rapid globalization, permanent change, and increasing risk.

Against this background, this paper assesses the current role of mindfulness in sustainability research, practice, and teaching.^3^ Using an extensive literature review complemented by an experimental learning lab (described in “Methodology”), we outline the core conceptual trajectories of mindfulness in general sustainability research, practice, and teaching (Sects. “Mindfulness in general sustainability research”, “Mindfulness in general sustainability practice”, “Mindfulness in general sustainability teaching”). We also assess it more specifically in relation to the field of climate change adaptation^4^ and risk reduction (Sects. “Mindfulness in climate change, adaptation, and risk reduction research”, “Mindfulness in climate change, adaptation, and risk reduction practice”, “Mindfulness in climate change, adaptation, and risk reduction teaching”). Finally, we discuss its potential role in sustainability science, providing a comprehensive framework for systematizing and analyzing related interlinkages, and highlighting related implications (theoretical, methodological, etc.).

## Methodology

The approach consisted of a literature review, which was complemented by an experimental learning lab on mindfulness in sustainability science, practice, teaching, and learning. The literature review included both grey literature and scientific papers (identified via Scopus, Web of Science, LUBsearch, and Google Scholar) that connected mindfulness and sustainability both explicitly and implicitly. To conduct a comprehensive review of relevant research across multiple disciplines, the search string included the following terms: (mindfulness OR mindful* OR contemplative OR compassion OR meditat*) AND (sustainability OR sustainable) AND/OR (“climate change adaptation” OR (adaptation AND climate) OR “risk reduction” OR “disaster response” OR “disaster recovery” OR “hazard mitigation” OR names of specific hazards, such as flood OR storm OR landslide OR earthquake). After screening the abstracts, irrelevant studies (i.e. false positives) were removed, while other significant studies were identified using snowball sampling of the references.^5^

Development of the experimental learning lab began in 2015. In 2016, it ran for 3 months and included 70 students from two sustainability-focused Masters’ Programs.^6^ The lab was incorporated into a course on sustainable planning, climate change adaptation, and risk reduction. Contemplative teaching and learning practices were integrated into required everyday course activities (reflecting, listening, debating, working together, etc.; “Appendix 1”). In addition, written assignments on sustainability and mindfulness were offered as graded tasks, and a total of 16 voluntary mindfulness sessions (“Appendix 2”) were conducted outside the usual course activities (i.e., lectures, seminars, group work, and field trips). The mindfulness sessions were implemented in coordination with the Students’ Health Centre, and related information was provided in the course schedule, the students’ course portal, and a closed Facebook group. The sessions lasted between 15 and 30 min and included a variety of techniques (“Appendix 2”). Written and oral course evaluations (response rates: 50/100%), and two surveys and a group discussion (response rates: 71/23/29%), were conducted to assess participants’ understanding and knowledge of mindfulness and sustainability, and the impacts of their mindfulness practices on learning^7^ (“Appendix 3”–“Appendix 6”). The first survey was conducted before the lab was implemented, while the second survey and the group discussion took place afterwards (“Appendix 3” and “Appendix 6”). The successful implementation of the experimental learning lab resulted in the development of a new Master’s level course on sustainability, inner transition, and mindfulness in 2016.^8^

The analysis of the data (literature and lab) involved: (1) the identification of patterns and core conceptual trajectories in current sustainability research, practice, and teaching, (2) a comparison of the review outcomes with the results of the experimental learning lab,^9^ and (3) a comparison of the identified patterns/ trajectories between the three areas (i.e. research, practice, and teaching) and an assessment of related implications (e.g. ontology, methodology, synergies, and research gaps). Literal reading and qualitative coding were used to analyze and triangulate the results (Glaser and Strauss 1967; Strauss and Corbin 1998).^10^

## Mindfulness and sustainability in research

This section presents the results of the general analysis of mindfulness and sustainability in research (Sect. “Mindfulness in general sustainability research”). Then, the mindfulness–sustainability relationship is analyzed in the specific context of sustainable climate change adaptation and risk reduction (Sect. “Mindfulness in climate change, adaptation, and risk reduction research”).

### Mindfulness in general sustainability research

The analysis identified three patterns (core conceptual trajectories) in mindfulness and sustainability research, namely:

- There is a blind spot in the academic debate on mindfulness in sustainability research.
- Research on mindfulness is increasing, which (implicitly) provides growing evidence of its positive effects and potential contributions to sustainability/ sustainability science.
- Only a few initial attempts have been made to examine mindfulness–sustainability linkages more explicitly.

On one hand, the literature review revealed that mindfulness is generally not addressed in sustainability research. Analyses focus on objective interactions between natural, social, and human systems, whilst subjective aspects of human beings tend to be ignored (Sumi 2007). The exceptions that were identified linked mindfulness to the human–nature connection and native ways of knowing (e.g., Anthony 2013; Lockhart 2011), social justice and social activism (e.g., Brown et al. 2007; Doetsch-Kidder 2012; Jacob et al. 2009), and more recently to sustainability-oriented innovations (Siqueira and Pitassi 2016; Lengyel 2015). This result was confirmed by the findings from the experimental learning lab. A total of 83% of participants said that they had not come across the issue of mindfulness in their environmental studies and sustainability science reading. In addition, only four respondents made links between mindfulness and nature/ the environment.

The fact that subjective human aspects tend to be ignored in sustainability research was confirmed in extensive reviews by Kjell (2011) and Kajikawa (2008), who independently found that sustainability and well-being research are two separate fields. Other scholars, such as Rinne et al. (2013) and Fabbrizzi et al. (2016), have also highlighted the lack of research at the intersection of societal sustainability and individual well-being. This gap can be illustrated by research into sustainable consumption and behavior. Kajikawa (2008) shows that studies on the topic generally focus on the impact of people’s consumption on sustainability, rather than the impact of aspects that lead to unsustainable consumption, such as lifestyles, well-being, or mindfulness (cf. Rogerson and Kim 2005).

On the other hand, mindfulness research is rapidly growing (AMRA 2016) and is making an increasing contribution to sustainability. Since 2009, there has been a 30% annual increase in the frequency of references to mindfulness in peer-reviewed science-, art-, and humanities-based articles (Ericson et al. 2014). The momentum is coming from fields such as psychology and medicine, which until recently have received minimal attention from sustainability practitioners and academics (Jones 2015). Such work focuses on a range of well-being and health-related conditions (psychological and physical) (Brown et al. 2007; Ericson et al. 2014; Davidson et al. 2003) and the activation of (intrinsic/ non-materialistic) core values (Sheth et al. 2010).

Although research into mindfulness and related attributes has not explicitly addressed the relationship between mindfulness and sustainability (Ericson et al. 2014), it has highlighted the complex linkages with sustainable development, from the individual to the global level (Brown et al. 2007). Derived from the principle of dependent origination, it recognizes that all beings are deeply connected to other beings and the world, including their actions and thinking (Yeh 2006).^11^ It recognizes the adaptive value of bringing consciousness to bear not only on subjective experience, but also on behavior and the environment (Amel et al. 2009; Brown et al. 2007; Carver and Scheier 1981; Csikszentmihalyi 1997; Duval and Wicklund 1972; Jacob et al. 2009).

In 2015, the notion of “ecological mindfulness” was put forward by sustainability scholars as a new approach to promote social and environmental sustainability (Mueller and Greenwood 2015; Sol and Wals 2015). This notion is based on research which suggests that mindfulness is associated with ecologically-responsible behavior that is oriented to the common good (Brown and Kasser 2005),^12^ although the specifics are culturally shaped (Chinn 2015). Ecological mindfulness also promotes the integration and blending of thought, rather than disintegration and separation (Mueller and Greenwood 2015). It can thus also be seen as an initial attempt to link the concepts of mindfulness and sustainability, as it lies at the intersection of ontological hybridity and can be seen as a way to approach the study of the world, or as a way to distance us from “either/ or” thinking, and move towards “not-only-but-also” thinking (Mueller and Greenwood 2015; Chadwick 2013). See also “Mindfulness in general sustainability practice” and “Mindfulness in general sustainability teaching”.

Whilst the literature review highlights that research on mindfulness and sustainability is scarce and fragmented, it provides scientific support for the positive influence of mindfulness on: (1) subjective well-being (e.g., Brown et al. 2007; Jacob et al. 2009; Khoury et al. 2013); (2) activation of (intrinsic/ non-materialistic) core values (e.g., Brown et al. 2007; Carmody et al. 2009; Brown and Kasser 2005; Shapiro et al. 2006; Sheth et al. 2010); (3) consumption and sustainable behavior (e.g., Amel et al. 2009; Brown and Kasser 2005; Brown and Ryan 2003; Brown et al. 2007, 2004; Ericson et al. 2014; Goleman 2009; Jacob et al. 2009; Sheth et al. 2010); (4) the human–nature connection (e.g., Amel et al. 2009; Anthony 2013; Howell et al. 2011; Lockhart 2011); (5) equity issues (e.g., Brown et al. 2007; Harris and Bordere 2016; Shah et al. 2012); and (6) social activism (e.g., Brown et al. 2007; Doetsch-Kidder 2012).^13^ Whilst these aspects are highly interlinked^14^ and clearly relate to wider (socio-political power) structures, mindfulness research tends to focus on the individual level.

### Mindfulness in climate change, adaptation, and risk reduction research

The analysis identified the following core conceptual trajectories in research that addresses mindfulness and sustainability in relation to climate change, adaptation, and risk reduction:

- There is a blind spot in the academic debate on mindfulness in anticipatory adaptation and risk reduction research.
- In the context of climate change and climate change mitigation, more studies can be found that link individuals’ state of being to sustainability.
- There is an increasing body of research on mindfulness in post-disaster response and recovery as a way to increase psychological resilience (with links to response and recovery preparedness).
- The concept of “organizational mindfulness” that was developed in the domain of risk and safety research has recently been applied to sustainability.

The literature review identified a blind spot in the academic debate on mindfulness in anticipatory adaptation and risk reduction research. This is supported by Ryan (2016) who states that there is a little research into the potential of enhanced emotional knowledge and well-being to prompt anticipatory adaptation.^15^ Here, “anticipatory” relates to the pro-active integration of adaptation and risk reduction in the pre-disaster context (development work), rather than the integration of such considerations in the post-disaster response (emergency assistance) or recovery (assistance for rehabilitation and reconstruction) (IPCC 2001, 2014). The results were confirmed by the findings from the experimental learning lab. A total of 83% of participants said that they had not come across the issue of mindfulness in the risk reduction and climate change adaptation literature.

In contrast, in the context of climate change and climate change mitigation, there are a growing number of studies that link individuals’ state of being to sustainability. In particular, the influence of emotional knowledge on how people experience and understand climate change is receiving increasing attention (Doherty and Clayton 2011; Koger 2015). However, only few studies have explored the influence of emotions, a powerful motivator for human behavior, on how individuals process and react to climate change information (Lu and Schuldt 2016). Exceptions are Ryan (2016) and Lu and Schuldt (2016). The latter explore how compassion influences individuals’ support for government actions to address climate change. They demonstrate that the influence of compassion extends beyond increasing the motivation to act in ways that alleviate immediate suffering, highlighting the overlooked role of mindfulness in contributing to citizens’ policy support regarding climate change mitigation.

Most of the identified studies that link mindfulness with climate change adaptation and/or risk reduction have examined mindfulness in the context of post-disaster response and recovery (with links to response and recovery preparedness). Doherty and Clayton (2011) confirm this result and also highlight the need to apply a better understanding of mindfulness in relation to disasters to the context of climate change. Nevertheless, current work mainly assesses the potential of specific mindfulness-related strategies and interventions for increasing psychological resilience in particular target groups, rather than individual mindfulness in general (i.e., mindfulness disposition) (Thompson et al. 2011). These interventions include meditation or relaxation techniques aimed at different groups, including children and young people affected by disasters (Catani et al. 2009; Zeller et al. 2015), disaster survivors and at-risk individuals (Hechanova et al. 2015; Hoeberichts 2012; Matanle 2011; Srivatsa et al. 2013; Yoshimura et al. 2015), disaster aid workers (Eriksen and Ditrich 2015; Hoeberichts 2012; Smith et al. 2011; Waelde et al. 2008), and disaster researchers (Eriksen and Ditrich 2015). The studies have advanced knowledge in relation to trauma/ traumatic stress reduction (see Thompson et al. [2011] for a review of related advancements). To date, cultural differences with respect to mindfulness and mindfulness interventions have barely been addressed (cf. Chinn 2015).

Recently, the notion of “organizational mindfulness” has emerged. The concept was developed in the domain of risk and safety research, and has only recently been extended to sustainability, and sustainable risk reduction in particular (Aviles and Dent 2015; Becke 2014; Becke et al. 2012; Senghaas-Knobloch 2014). Weick and Sutcliffe (2007) based their conceptualization of organizational mindfulness on high-reliability organizations (i.e. organizations that must find effective ways of dealing with potential catastrophes resulting from the inherently complex and dangerous nature of their work [cf. Sutcliffe 2011]).^16^ The concept highlights collective and organizational learning with respect to the anticipation of, and coping with, unexpected risky events that are found in volatile and unpredictable environments, and are harmful to the viability of organizations (Becke et al. 2012; Becke 2014). In addition, organizational mindfulness refers to the idea that actively nurturing and developing social resources is key to organizations’ longevity and sustainability, and especially critical for organizations facing extreme events with potentially long-lasting consequences (Becke 2014).

Overall, the literature review highlights that the field is still emerging. In addition to aspects related to sustainability in general, studies particularly highlight and provide scientific support for the positive influence of mindfulness on: (1) minimizing automatic, habitual, or impulsive reactions; (2) facilitating more flexible, adaptive responses to events (e.g., Brown et al. 2007; Hechanova et al. 2015; Waelde et al. 2008); and (3) influencing individuals’ support for planned actions to address climate change (all of which are relevant to the anticipation of, and coping with, unpredictability in organizations [i.e., “organizational mindfulness”]). Here, ‘planned’ adaptation is the result of a deliberate (governmental) policy decision, based on an awareness that conditions have changed—or are about to change—and that action is required to return to, maintain, or achieve a desired state (IPCC 2001, 2014).

## Mindfulness and sustainability in practice

This section presents the results of the analysis of mindfulness and sustainability practice in general (Secti “Mindfulness in general sustainability practice”). It is then analyzed in the specific context of climate change adaptation and risk reduction (Secti “Mindfulness in climate change, adaptation, and risk reduction practice”).

### Mindfulness in general sustainability practice

The literature review revealed the following core conceptual trajectories:

- Mindfulness-based responses to environmental challenges are being increasingly promoted.
- Notions such as the “mindfulness revolution”, “contemplative environmental practice”, “contemplative practice for sustainability”, and “ecological mindfulness” have emerged.

In practice, mindfulness-based responses to environmental challenges are increasingly promoted by development organizations, networks, and coalitions; sometimes termed the “mindfulness revolution”. This refers to the rapid emergence of initiatives and literature that aim to revolutionize current sustainability practice (Boyce 2011; Edwards 2015; Koger 2015). This result is in line with the outcomes of the experimental learning lab, where participants who had come across mindfulness in their readings referred to the practice-related approaches found in green movements.

Mindfulness-based responses to environmental challenges are promoted by both secular and faith-based organizations, and provide support for individuals and institutions (Edwards 2015; Koger 2015).^17^ They encourage mindful awareness of underlying emotions, thoughts, values, and experiences that contribute to (un)sustainable actions, in turn, leading to increased social activism and justice (Hanh and Weisman 2008; Kaza 2008).^18^

In this context, notions such as “contemplative environmental practice”, “contemplative practice for sustainability”, and “ecological mindfulness” have emerged (cf. Sects. “Mindfulness in general sustainability research” and “Mindfulness in general sustainability teaching”). They are increasingly promoted by all kinds of organizations, including private businesses, non-profit, and faith-based organizations (AASHE 2016; Sangha 2016b; Unlimited 2015). Although implicit, applications often relate to the issue of climate change mitigation (cf. Sect. “Mindfulness in climate change, adaptation, and risk reduction practice”). An example is the Whidbey Institute, which, in cooperation with the Washington Centre, organized a conference in 2014 on the issue of “sustainability and contemplative practice” (see also “Mindfulness in climate change, adaptation, and risk reduction practice” and “Mindfulness and sustainability in teaching”). Other potential areas of application include the eco-tourism sector (Lengyel 2015).

### Mindfulness in climate change, adaptation, and risk reduction practice

The analysis identified the following core conceptual trajectories with respect to mindfulness in relation to climate change, adaptation, and risk reduction practice:

- Many faith-based organizations recognize the need to respond to climate change and provide mindful-based direction for that response.
- There is also an increase in secular initiatives that promote mindfulness-based methods to respond to climate change.
- Most mindfulness-related practice relates to climate change mitigation, rather than climate change adaptation.
- Exceptions relate mostly to the promotion of mindfulness-based response and recovery by different emergency organizations, including preparedness.

Many faith-based organizations, including leading Buddhist and Christian groups (e.g., the Vatican), are recognizing the need to respond to climate change, and are asked to provide mindful-based direction to entities such as the United Nations, governmental and non-governmental institutions (Koger 2015).^19^ In 2014, the United Nations Framework Convention on Climate Change (UNFCCC) requested, for instance, the Buddhist leader Thich Nhat Hanh to provide a statement on climate change. This was subsequently published on the UNFCCC website ahead of the Paris Climate Summit in September 2015 (Hanh 2015). Hence, the faith community is also playing an increasingly important role in holding governments accountable for mindfully responding to climate change and addressing climate justice (Koger 2015; Sangha 2016a). Influential groups include GreenFaith (led by Christians and Jews) and the (Buddhist) One Earth Sangha, which published a series of online conversations on “Mindfulness and Climate Action”, and the Dharma Teachers Statement on Climate Change (Dharma Teachers International 2014). Other bodies include the Convergence Community, a global network of religious–environmental leaders, and the Our Voices coalition that was specifically created to bring faith to the Paris Summit. Joint efforts by these actors resulted in an Interfaith Statement on Climate Change that was published in response to the Paris Agreement.^20^ Notably, the faith community is also an important driver of public opinion and mindful actions taken in response to climate change. A recent study has, for instance, demonstrated the so-called “Francis effect”, i.e., the positive effect that Pope Francis and his encyclical “Laudato Si: On Care for Our Common Home” has had on people’s perceptions and responses to climate change (Maibach et al. 2015) (see footnote 17).

In addition, there are an increasing number of secular initiatives that promote mindfulness-based methods to support both individuals (including sustainability and environmental professionals) and organizations in promoting the transition to a more climate-resilient society (Koger 2015). However, most of these initiatives relate to climate change mitigation, i.e., the reduction of greenhouse gas emissions. In this context, the term “mindful climate action” was coined.^21^ Examples are “Active Hope” as well as the “Work that Reconnects Network” and other initiatives based on deep ecology pioneer Joanna Macy’s perspective on ecological activism (Macy and Young Brown 1998; Vaughan-Lee 2013).

In contrast, there is less evidence of mindfulness-related practice in the fields of climate change adaptation and risk reduction, and there is hardly any evidence of mindfulness-related practice that is explicitly focused on anticipatory adaptation and risk reduction.

However, consistent with existing research (cf. Sect. “Mindfulness and sustainability in research”), mindfulness-based approaches to disaster response and recovery are increasingly promoted, especially by emergency organizations. One example is the Red Cross, who developed an “After the emergency” podcast for young people affected by the 2009 Victorian bushfires. The podcast provides information about trauma, how to cope with the stress of an emergency, and how to increase psychological resilience in the long term (Australian Red Cross 2015).

These results contrast with the outcomes from the experimental learning lab. Whilst a total of 79% of respondents felt that mindfulness had an influence on their daily life in terms of sustainable behavior, 32% thought that it was irrelevant to sustainability practice, in general, and adaptation and risk reduction in particular.

## Mindfulness and sustainability in teaching

This section presents the results of the analysis of mindfulness in sustainability teaching, in general (Sect. “Mindfulness in general sustainability teaching”). It is then analyzed in the context of teaching sustainable climate change adaptation and risk reduction (Sect. “Mindfulness in climate change, adaptation, and risk reduction teaching”).

### Mindfulness in general sustainability teaching

The analysis revealed the following core conceptual trajectories:

- Compared to pedagogy in general, mindfulness has received little attention in sustainability teaching and learning.
- Contemplative methods have only recently been explicitly promoted as a new way of teaching and learning that is needed to create a more sustainable society.
- In line with this, the notion of “ecological mindfulness” has emerged to promote a different way of learning and foster scientific understanding and action.
- Recently scholars have argued for the need for mindfulness in improving sustainability institutions and curricula.

Mindfulness is increasingly recognized and used in pedagogy (Black 2010; Schoeberlein 2009; Schonert-Reichl and Roeser 2016). Despite an increasingly fragmented educational discourse in general (Mueller and Greenwood 2015; Sameshima and Greenwood 2015), it is receiving mainstream acceptance as a way to enhance both students’ and teachers’ well-being (e.g., Albrecht et al. 2012; Black et al. 2009; Greenberg and Harris 2012; Mendelson et al. 2010). Its success is based on a wealth of research that supports the benefits of mindfulness for memory, learning, emotional regulation, and well-being, together with its importance for interpersonal and emotional aspects of pedagogy and the teaching environment (e.g., Biggs and Tang 2011; Hülsheger et al. 2013; Illeris 2009; IMS 2015; Lee 2012; Meiklejohn et al. 2012; Wisner 2014). The results from the experimental learning lab indicated that 60% of survey participants felt that mindfulness was relevant for sustainability teaching and learning (pre-lab survey), which increased to 79% after the lab ended. In addition, those who had participated in the voluntary mindfulness sessions agreed that it had a positive influence on their learning.

In recent years, scholars have turned their attention to defining theoretical models for mindful teaching, and their translation into pedagogical practice (e.g., Albrecht et al. 2012; Ragoonaden 2015; Weaver and Wilding 2013).^22^ Mindful teaching is seen as an approach that integrates the following aspects: (1) the building of a “community” or connection (teacher–student and student–student) based on compassion, non-judgmental, and accepting openness, and the establishment of respectful boundaries; and (2) the creation of an engaging and reflective learning environment, which supports self-observation and mutual learning, whilst acknowledging differences in cultural backgrounds, experiences, social behavior, and learning (Wamsler 2015/2016).

While mindfulness is playing an increasing role in pedagogy, in general, it has received limited attention in the context of sustainability teaching and learning. It is only recently that contemplative teaching methods have explicitly been promoted as a new way to address socio-ecological challenges and create a more just, compassionate, reflective, and sustainable society (ACMHE 2016; Gugerli-Dolder and Frischknecht-Tobler 2011; Gugerli-Dolder et al. 2013; Litfin and Abigail 2014; Schoeberlein 2009). This is seen in the recent increase in organizations and institutions that offer workshops, seminars, professional networks, and training on the subject.^23^

As in general sustainability research and practice (Sects. “Mindfulness in general sustainability research” and “Mindfulness in general sustainability practice”), “ecological mindfulness” is emerging in sustainability teaching (Mueller and Greenwood 2015; Sol and Wals 2015). Underlying this notion is the idea that the proliferation of “adjectival education”^24^ (including sustainability education) is inconsistent with the interdisciplinary and cross-hybrid learning needed to foster scientific and cultural understanding and actions leading to socio-ecological change. Hence, ecological mindfulness suggests that the integration and blending of thought, rather than its disintegration and separation, should be the purpose of sustainability teaching and learning (Mueller and Greenwood 2015; cf. Sects. “Mindfulness in general sustainability research” and “Mindfulness in general sustainability practice”). Furthermore, scholars argue that the ecological mindfulness of teachers is crucial in shaping students’ understanding of nature–society relations, and that it requires integrating indigenous cultural knowledge and sustainable practices within existing scientific frameworks (Chinn 2015).

In addition, scholars have recently argued for the need for mindfulness approaches to improve educational bodies and curricula oriented towards sustainability and well-being (e.g., linked to the notion “ecological learning”). It is argued that in the context of sustainability, teaching and learning require spaces where diverse ecological, holistic, and place-responsive perspectives can take root, be nurtured, and flourish into ways of knowing, being, and becoming that serve people, places, and the planet (Greenwood 2013; Gugerli-Dolder and Frischknecht-Tobler 2011; Sameshima and Greenwood 2015). In addition, teaching should become a way to work towards a “learning system”, in which people collectively become more capable of withstanding setbacks and dealing with insecurity, complexity and risks, in which mindfulness can play a role (Sol and Wals 2015).

### Mindfulness in climate change, adaptation, and risk reduction teaching

The analysis revealed the following core conceptual trajectories:

- Contemplative teaching and learning methods are being explored in the context of sustainability education, notably to address new demands caused by climate change (i.e., individual capacities and qualities).
- In contrast, there is a little academic discourse on contemplative methods for climate change adaptation and risk reduction education.
- There is, however, an increase in neuroscience-based mental health and mindfulness training provided by private institutions to help people to cope with climate-enhanced adversities.

In light of the growing risk and uncertainties, sustainability is increasingly being referred to as a learning challenge. It is argued that in addition to appropriate forms of governance, legislation, and regulation, alternative forms of education and learning are needed for people to develop capacities and qualities that allow them to contribute to alternative (climate adapted) behaviors, lifestyles and systems, both individually and collectively (Sol and Wals 2015).

Consequently, contemplative teaching and learning methods are being explored in sustainability education, particularly regarding courses that address climate change issues. Examples are the revision and development of new syllabuses on global environmental politics, sustainability leadership development and “mindful climate action” (Barret et al. 2016; Litfin and Abigail 2014).^25^ In line with this, 79% of the survey participants in the experimental learning lab felt that mindfulness was relevant to sustainability teaching and learning, including issues of climate change adaptation and risk reduction, while those who had participated in the mindfulness sessions agreed that they had had a positive influence on related learning. Overall, around 80% welcomed the integration of mindfulness into the course, and 20% were neutral (based on the pre-lab survey and oral course evaluation). Around 64% stated that the lab added extra value to the course in general. Only 1 out of 70 students said that its continuation would not be worthwhile (oral course evaluation).

However, there is a little academic discourse on the subject of contemplative adaptation and risk reduction education, although such topics are very sensitive and can trigger memories of grief, sorrow and vulnerability (Wamsler 2015/2016). This contrasts with an increase in neuroscience-based mental health science and mindfulness training offered by private organizations to assist people (including students and professionals) to cope with and address climate-enhanced adversities (cf. Sect. “Mindfulness and sustainability in practice”). One example is the International Transformational Resilience Coalition and the program offered by The Resource Innovation Group in partnership with Resilience Training International and the Trauma Resource Institute that aims to enhance personal, collective, and environmental well-being (Doppelt 2016).

## Discussion and conclusions: Integrating mindfulness into sustainability research, practice, and teaching

The results of this study show that there is a theoretical, conceptual, and empirical blind spot in the academic debate on mindfulness in sustainability research, practice, and teaching.^26^ This is alarming, since sustainability encompasses not only ecological and economic, but also social dimensions at all scales. Sustainability is ultimately a social choice. It is about what to develop, what to sustain, and for how long (Parris and Kates 2003), and is thus also a deeply normative process (Kemp and Martens 2007). Consequently, individual and subjective modes of being, such as mindfulness, play a crucial role in the context of the scientific inquiry, practice, and teaching of sustainability.

Current knowledge on mindfulness in sustainability is both scarce and fragmented; however, it is gaining increasing momentum. The field is only just emerging; nearly all of the relevant literatures has been published in the past 5 years. While there appears to be increasing consideration of mindfulness in sustainability research, practice, and teaching, most is related to practice.

In research, most progress relates to mindfulness in reactive adaptation and risk reduction during disaster response and recovery. Related work focuses on agency-based solutions, but does not address how this could be translated into structural, systemic change. Little attention is given to pro-active adaptation and risk reduction^27^ and sustainability science in general, related scientific inquiry and methods.

In practice, most progress has been made in the field of climate change mitigation. Mindfulness approaches are based on compassion and positive emotion, unlike the “motivation by fear” and “crisis approach” strategies often found in climate change communications and responses (Ryan 2016). In contrast, little explicit consideration is given to sustainability practice in general, and anticipatory adaptation and risk reduction practice in particular.

In education, most progress relates to an increased recognition of contemplative teaching, although there is no explicit consideration given to the domain of sustainability science (including adaptation and risk reduction). This is in stark contrast to the potential role of science education in mediating the structure and function of the brain to support sustainable change (Powietrzynska et al. 2015).

The literature review did not find any structural critiques of the potential drawbacks of mindfulness in the specific context of sustainability (in research, practice, and education). Nevertheless, there are critiques regarding mindfulness, in general. Concerns have been voiced about potential side-effects (Howard 2016), the inappropriate use of techniques (Williams and Kabat-Zinn 2011; Purser and Loy 2013), and the potential co-optation of mindfulness for capitalist purposes (Carrette and King 2005), stripping it of its transformative power. A reflexive approach to sustainability is key to addressing such concerns in research, practice, and teaching.

**Fig. 1:**
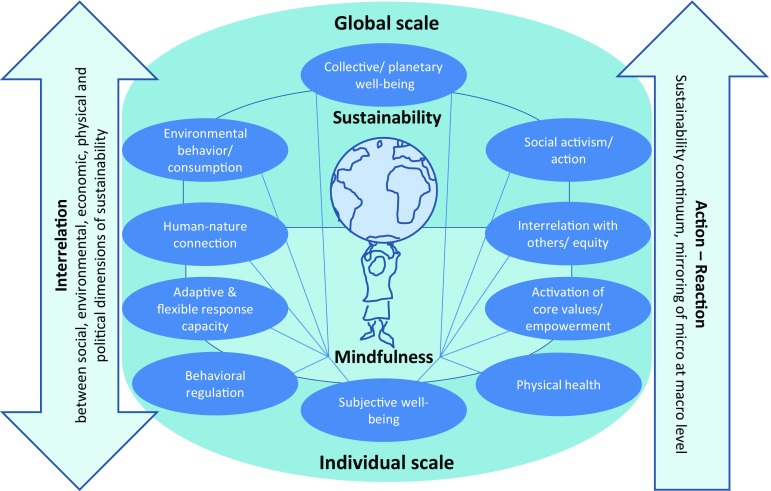
Framework for contemplative scientific inquiry, practice, and education in sustainability *Source*: Developed/ designed by Wamsler, C. Note that the figure does not imply a distinction/ categorization between the right- and left-hand side aspects

This study identified key aspects relevant to mindful inquiry, practice, and education in sustainability, which were developed into a framework for systematizing and analyzing the interlinkages between mindfulness and sustainability from the individual to the global scale (Fig. 1). These aspects are culturally shaped and include: (1) subjective well-being; (2) activation of (intrinsic/ non-materialistic) core values; (3) consumption and sustainable behavior; (4) the human–nature connection; (5) equity issues; (6) social activism; and (7) deliberate, flexible, and adaptive responses to climate change. The framework thus supports the understanding that mindfulness can be seen as a key concept to politically sensitizing people and organizations to the consequences of unquestioned structures and power relations (cf. Senghaas-Knobloch 2014 and “Mindfulness in general sustainability practice”). The mindfulness-sustainability framework can broaden the spatial horizon and help to understand impacts on (distant) communities that might be incongruent with declared values. Understood in this way, mindfulness is no longer a concept that only addresses cognitions and cognitive schemes, but also fosters a sense of appropriate or just behavior (cf. Senghaas-Knobloch 2012). It, therefore, bridges the gap between individual and global scales and wider socio-political structures (cf. Fig. 1, Sects. “Mindfulness in general sustainability research” and “Mindfulness in general sustainability practice”).

The framework positions mindfulness within sustainability science, which may result in more nuanced understandings and perceptions, inspire action, and enhance sustainable change. It may lead to more expansive and inclusive research and (writing) methods that enable people to take risks—the kind of risks that cannot be taken when academic fiefdoms determine the questions that are asked and regulate methodologies, rather than encourage creativity (cf. Mueller and Greenwood 2015).

Science has always been shaped by current problems, and it evolves with them. Climate and disaster risk is global, complex, pervasive, and a new subject of scientific inquiry. Until now, reductionist, natural science research has been taken as the intellectual and social model. However successful, it has been in the past, emerging policy issues and research on neuroplasticity, emotions and mindfulness show that this ideal of rationality is no longer appropriate. Recent developments raise questions about the ontological frameworks and the materialist paradigm that shape the construction of knowledge in general (Osborne and Grant-Smith 2015; Schwartz 2011), and sustainability knowledge and science in particular. Non-material causation need to be recognized as part of sustainability (cf. Sect. “Mindfulness in general sustainability research”).

Hence, theory and research on both sustainability and mindfulness would benefit from synergies towards sustainable change. On one hand, mindfulness research can enhance sustainability science by better linking all scales: from the individual to the global, and advancing ontological questions of scientific inquiry. On the other hand, sustainability science can enhance mindfulness research. In particular, it makes it possible to go beyond agency-based approaches, and examine interdependencies, related power issues, and mindfulness from a decontextualized perspective. In this context, it is important to recognize cultural differences (cf. Christopher et al. 2008, 2009; Kabat-Zinn 2003), which can be embraced through transdisciplinary approaches that encourage cross-cultural exchanges and create new understandings of sustainability (Chinn 2015).

While there are still many unanswered questions in the separate fields of sustainability and mindfulness, reconciling the two areas may open up opportunities for a more profound understanding. Sustainability requires an understanding of causes, consequences and the dynamics of a holistic, interdependent form of well-being in which mindfulness (and associated emotional intelligence) is an important aspect. Rather than looking at active mindfulness interventions and how they play out (e.g. Jacob et al. 2009), further research should also look at individual mindfulness disposition and link it to sustainability. This would open the way for a broader discussion on the role of mindfulness, inner transition, and spirituality in general, in sustainability. It excludes the isolated consideration of individual aspects (e.g., empathy) and erroneous applications of mindfulness that are also deployed to support consumerist values and capitalism that lie at the root of unsustainability (Becker 2015; Weil 2016; Williams and Kabat-Zinn 2011).

We conclude that mindfulness can contribute to understanding and facilitating not only individual, but societal sustainability at all scales. It should, therefore, be considered as a core concept in sustainability research, practice, and teaching. We end with a call for more sustainability research that acknowledges positive emotional connections, spirituality, and mindfulness in particular, recognizing that the micro and macro are mirrored and interrelated.

## Appendices: Experimental learning lab

### Appendix 1: Contemplative teaching and learning practices

The course director (and main author of this article) is a member of the Association for Contemplative Mind in Higher Education (ACMHE) with experience in integrating contemplative teaching and learning practices into postgraduate courses on sustainability, disaster risk reduction, and climate change adaptation. In the context of teaching sustainability science, students’ general (increasing) stress levels (see Medin and Lindberg 2013) are coupled with their emotional discomfort with respect to sustainability challenges (ecological depletion, climate change, etc). In addition, topics such as sustainable disaster risk reduction and climate change adaptation can trigger memories of grief, sorrow, and vulnerability. Fostering mindful interactions between the teacher and students, and among students, was thus seen as an important aspect of both successful teaching and learning, especially since these topics were taught to extremely diverse groups of students, from a multitude of backgrounds, nationalities, and disciplines and who have huge variations in age and experience.

Mindful teaching and supervision means showing compassion, with and for the students, fostering respectful and non-judgmental interaction, and increasing awareness, observation, and reflection on relevant subjects. Furthermore, it involves the creation of mindful interactions and the co-production of knowledge, which the course director supported both by implicit and explicit interventions.

The course director used the following opportunities to implicitly weave in mindful interaction:

- At the beginning of the course, the importance and value of every single person (students and teachers) and their responsibility in jointly developing knowledge and mutual trust were stressed.
- Students were supported to interact and get to know each other in mindful ways (e.g., when going through the attendance list, by not calling out their names but letting them group themselves based on shared interests and states of minds).
- Other interactive elements (icebreakers) were used to make students feel seen and welcome, and stimulate positive and meaningful conversation.
- In both teaching and supervision, conversations were steered away from judgmental opinions or gossip.
- An extra-curricular activity was offered alongside the course to provide more room for mindful and diverse forms of teacher–student and student–student interaction.

In addition, the course director integrated mindfulness-based approaches more explicitly in the following ways:

- Moments of silence and reflection were incorporated in course activities to improve self-reflection, self-awareness, social regulation, and empathy (cf. Goleman 2011).
- Mindful interactions during listening, debating, reflecting, and working together were explicitly encouraged during lectures, exercises, and seminars.
- The literature seminar included a written reflection on students’ learning in relation to the five key aspects of mindfulness: observing, describing, acting with awareness, non-judgement, and reactivity (cf. Baer et al. 2006).
- Group assignments required the students to establish rules for mindful interaction and learning.
- Written assignments on the topic of sustainability and mindfulness were offered as graded tasks. More specifically, in the context of the overall theme of the assignment (i.e., urban and/or rural sustainability, with a focus on risk reduction and adaptation planning), the groups were free to select a specific topic (including gender, livelihoods, food security and farming, municipal governance, climate networks, climate change mitigation, living labs, nature-based solutions, city-citizen cooperation, citizen participation, sectoral mainstreaming, or mindfulness). Two out of 14 groups decided to work on the issue of mindfulness: one implicitly (in the context of local indigenous knowledge), one explicitly (McDonald et al. 2016).
- Voluntary mindfulness sessions were offered and conducted outside the usual course activities (see “Appendix 2”).

For more information, see Wamsler (2015/2016).

### Appendix 2: Voluntary mindfulness sessions

The following mindfulness techniques were included in the experimental learning lab:

- Headspace. Web-based guided meditation techniques for mindfulness recommended by the Student Health Center. http://www.headspace.com.
- Mindful walking. http://tinyurl.com/zwxv3kl; http://www.chopra.com/articles/mindful-walking-practice-how-to-get-started. Kabat-Zinn (1994/2005).
- Deep listening. http://www.mindful.org/deep-listening. Gibson and Wisner (2016)
- Free writing. McKinney (1976).
- Gratitude meditation. O’Leary and Dockray (2015).
- Raisin/ concentration exercice. http://hfhc.ext.wvu.edu/r/download/114469. Weger et al. (2012).
- Mindful breaks and use of mindful bells. http://www.mindfulnessdc.org/bell.
- Self-compassion. http://www.mindfulselfcompassion.org/meditations_instructions.php. http://www.self-compassion.org. Neff and Germer (2013).
- 3 min breathing space. Short meditation. http://www.mindful.org/the-three-minute-breathing-space-practice. http://www.oxfordmindfulness.org/learn/resources.

One mindfulness session was facilitated by staff from the Student Health Centre, and the other sessions were facilitated by the four co-authors of this paper, supported by visual and Web-based aids. The sessions were integrated in the course schedule and built on each other:

**Table:**

Course week 1	Introduction of the experimental learning lab
Course week 2	Headspace introduction Headspace I (video and exercise) Headspace II (video and exercise) Mindfulness introduction by the Student Health Centre (including 3 min breathing space and raising/concentration exercise)
Course week 3	Headspace III (video and exercise) Headspace IV (exercise) Free writing
Course week 4	Gratitude mediation Headspace V (video and exercise) Headspace VI (exercise) and short mid-term feedback session
Course week 5	Mindful breaks Use of mindful bells Mindful walking 3 min breathing exercise (Facebook page) Headspace VII (video and exercise)
Course week 6	Self-compassion Deep listening
Course week 7	Headspace VIII (exercise—skipped due to time constraints) Headspace IX (video and exercise)
Course week 8	Headspace X Self-compassion Reflection exercise and oral evaluation

Attendance ranged from a maximum of 39 to a minimum of seven (due to the Easter break), with an average of 19.4 (including student facilitators and the course coordinator). The students that did not participate struggled with the scheduling, which was a major obstacle (reported during the oral course evaluation with 100% participation)

### Appendix 4: Oral ex-post course evaluation

Questions:

- How did you experience the experimental learning lab?
- How would you evaluate the experimental learning lab?
- Would it be of interest/of relevance to continue with the experimental learning lab activities?

Extract of oral course evaluation regarding the integrated experimental learning lab (100% participation): The interactive learning lab on mindfulness in sustainability science, practice, and teaching worked out very well. The separate mindfulness sessions were attended by around one-third of the class. Only one student said that he/she would recommend discontinuation. The other students were either positive or neutral. On this basis, it was agreed that the issue of mindfulness should be further integrated into future course activities.

### Appendix 5: Written ex-post course evaluation

Questions included in the overall course evaluation on the experimental learning lab (50% participation):

## Footnotes

1 Research has shown that emotional intelligence (including self-awareness, self-regulation, social awareness, and relationship management) can be increased through mindfulness practice and associated greater arousal of the brain’s left hemisphere (Goleman 2011).

2 For instance, theories of reflective self-consciousness and integrative awareness (Brown et al. 2007).

3 In this paper, ‘sustainability’ describes the meeting point of ideas, policies, and science that address challenges arising from the interaction of natural and social systems to create conditions under which humans and nature can exist in productive harmony to support present and future generations (cf. Scoones 2007). Accordingly, sustainability science seeks to investigate nature–society interactions and to identify creative solutions for more sustainable pathways, by reconciling natural and social sciences and supporting science–policy integration (Kates et al. 2001; Jerneck et al. 2011).

4 In this paper the terms climate change adaptation, climate adaptation and adaptation are used as synonyms.

5 The used search string resulted in a considerable number of papers to be reviewed (e.g., 610 in Scopus). However, as the mindfulness–sustainability field is still emerging, there were a large number of false positives. The percentages of empirical versus theoretical/ review papers regarding mindfulness and sustainability in research were around 50–50.

6 The two Masters’ Programs are the Lund University International Master’s Program in Environmental Studies and Sustainability Science (LUMES), run by Lund University Centre for Sustainability Studies (LUCSUS), and the International Master’s Program in Disaster Risk Management and Climate Change Adaptation, run by the Department of Risk Management and Societal Resilience. Mindfulness approaches were first integrated into the course design in 2013. Their expansion and subsequent development into a learning lab was initiated in 2015 following discussions with the Pedagogic Academy and a LUMES “Knowledge to Action” student project.

7 Mainly in relation to the five key aspects of mindfulness established by Baer et al. (2006): observing, describing, acting with awareness, non-judgement, and reactivity, together with related aspects of emotional intelligence established by Goleman (2011): self-awareness, self-regulation, social awareness (including empathy, and compassion) and relationship management.

8 Original title: Sustainability, Mindfulness, and Compassion.

9 Due to the explorative and qualitative character of this study, there were no control groups. In theory, students who did not participate in the mindfulness sessions could have been used as the control group. However, the response rates were too low.

10 Based on the research setting, some basic organizational categories were established prior to the data review (categorizing strategy–open coding). Within these primary ‘bins’, patterns were identified (pattern matching–axial coding). The final step was the identification of connections and relationships through a comparison of different categories and patterns (theory building–selective coding) (cf. Wamsler 2007).

11 See also Egli (1994) for a discussion on the action–reaction relationship between individual and global scales.

12 This implies a state of mindfulness where people become more aware of environmental impacts and their causes, and adjust their behavior accordingly.

13 Further research is however called for to look into causality of mindfulness and related methodological challenges, which was outside the scope of this research. Further note that supporting references include empirical studies or systematic reviews of studies that report empirical data (e.g., Khoury et al. 2013). .

14 For example, studies have identified positive feedback loops between: (1) mindfulness, subjective well-being, equity issues, and social activism (e.g. Shah et al. 2012); (2) mindfulness, the human–nature connection, subjective well-being and pro-environmental behavior (e.g. Barbaro and Pickett 2016); and (3) mindfulness, intrinsic values, subjective well-being, and pro-environmental behavior (e.g. Brown and Kasser 2005).

15 An exception is Lyles (2015) who looks into the potential of applying compassion building programs for sustainable planning in general, and risk reduction and adaptation planning in particular.

16 Mindfulness is increasingly advocated in the context of different types of businesses and in organizational learning in general (e.g., Schley 2011; Senge et al. 2006; Townsend 2013).

17 For example, the International Society of Sustainability Professionals, EcoSTEPS (Crawford 2013).

18 See “Mindfulness in general sustainability research” and Yeh (2006) for related discussions on the principle of dependent origination and its link to sustainable development. For further discussions on mindfulness as a politically sensitizing concept see Senghaas-Knobloch (2012, 2014), Becke (2014) and Becke et al. (2012).

19 Mindfulness, mental focus and contemplation are associated with many religions and spiritual traditions. For a discussion on mindfulness in Christianity see, for instance, Hanh (2000).

20
http://www.interfaithstatement2016.org/read_the_statement.

21 For example, https://vimeo.com/116373704.

22 Related teaching approaches also use the concepts/ terms “engaged teaching” (e.g., Weaver and Wilding 2013) and “compassionate teaching” (e.g., Wolpow et al. 2009) and “contemplative education” (e.g., Zajonc 2016).

23 For example, the Association for Contemplative Mind in Higher Education (ACMHE) that was founded in 2008.

24 Adjectival education refers to the segmentation of knowledge fields (including sustainability science) into separate domains.

25 At the University of Wisconsin, a “Mindful Climate Action” education program was, for instance, designed to help decrease carbon footprints while enhancing personal health and happiness.

26 “Theoretical” refers to the foundational theories and associated research that link mindfulness and sustainability, while “conceptual” refers to their operationalization into frameworks for research, practice and teaching.

27 Note that Collins (2015) highlighted the need for learning and decision making in risk reduction planning based on both experience, and intuition in order to capture imaginative responses that might be supported by mindfulness (cf. Dane 2011; Remmers et al. 2015). This is for instance an area that has not, so far, been explored in any detail.
